# Neutralizing monoclonal antibodies for early treatment of hospital‐acquired SARS‐CoV‐2 infection in hematologic patients

**DOI:** 10.1002/jha2.554

**Published:** 2022-09-02

**Authors:** Linda Bussini, Diletta Testi, Beatrice Tazza, Chiara Oltolini, Sara Mastaglio, Chiara Sepulcri, Caterina Campoli, Filippo Trapani, Zeno Pasquini, Emanuela Zappulo, Matteo Bassetti, Pierluigi Viale, Malgorzata Mikulska, Michele Bartoletti

**Affiliations:** ^1^ Infectious Diseases Unit, IRCCS Azienda Ospedaliero‐Universitaria di Bologna Policlinico di Sant'Orsola Bologna Italy; ^2^ Department of Medical and Surgical Sciences Alma Mater Studiorum University of Bologna Bologna Italy; ^3^ Department of Infectious Diseases San Raffaele Scientific Institute Milano Italy; ^4^ Department of Hematology and Bone Marrow Transplantation Unit San Raffaele Scientific Institute Milano Italy; ^5^ Department of Clinical Medicine and Surgery University of Naples Federico II Napoli Italy; ^6^ Department of Health Sciences (DISSAL) University of Genoa Genoa Italy; ^7^ Infectious Diseases Unit IRCCS Ospedale Policlinico San Martino Genoa Italy

**Keywords:** anti‐SARS‐CoV‐2 monoclonal antibodies, COVID‐19, hematologic malignancy

## Abstract

Efficacy of early treatment with anti‐SARS‐CoV‐2 spike protein monoclonal antibodies (mAbs) for nosocomial SARS‐CoV‐2 infection in hematologic patients is unknown. Retrospective, cohort study conducted in four Italian teaching hospitals. We included adult patients with hematologic malignancies and hospital‐acquired SARS‐CoV‐2 infection diagnosed between November 2020 and December 2021. The principal exposure variable was administration of mAbs. The primary endpoint was clinical failure dea composite outcome of mortality and/or invasive and noninvasive ventilation within 90 days from infection onset. We included 52 patients with hospital‐acquired SARS‐CoV‐2 infection. Males were 29 (60%), median age was 62 (interquartile range [IQR] 48–70). Forty‐five (86%) patients were on chemotherapy or had received chemotherapy within 30 days. MAbs were administered in 19/52 (36%) patients. Clinical failure occurred in 22 (42%) patients; 21% (4/19) in mAbs group versus 54% (18/33) in non‐mAbs group (*p* = 0.03). Other predictors of clinical failure were older age (median [IQR] 69 [61–72] versus 58 [46–66], *p* = 0.001), and higher Charlson comorbidity index (median [IQR], 5 [3.25‐5] versus 3 [2–5], *p* = 0.002). At multivariable Cox regression model, mAbs were independently associated with a significantly lower rate of clinical failure (HR 0.11, 95% CI 0.01–0.85, *p* = 0.01), after adjusting for confounders. In conclusion, mAbs are promising for early treatment of hematologic patients with healthcare‐related SARS‐CoV‐2 infection.

## 1 INTRODUCTION

Compared to general population, patients with active hematologic malignancies (HMs) who acquire SARS‐CoV‐2 infection develop more often severe or critical Coronavirus 2019 Disease (COVID‐19): 15% versus 62%, respectively [1]. Although improvement of diagnosis and therapies of COVID‐19 had reduced mortality from the beginning of pandemic, data from the OnCovid registry—the European registry of adult patients with solid or hematologic cancer and COVID‐19—still showed a far higher case‐fatality rate than general population, reaching 14% [2]. In addition, current literature reports that patients with HMs have a higher and more prolonged viral shedding [3]. All these characteristics affecting the course of COVID‐19 may delay the access to chemotherapy or transplantation, consequently affecting the prognosis of the hematologic disease. Finally, efficacy of vaccination in preventing severe disease could be lower in this population, since the impaired immunity may not guarantee the development of protective antibody titres [4]. Moreover, patients with active HM may frequently need hospitalization for undeferrable chemotherapy. However, epidemiologic data have shown that more than one third of immunocompromised patients acquired SARS‐CoV‐2 infection during a hospitalization, thus, nosocomial infections have been constantly increasing since the beginning of pandemic with potentially catastrophic consequences [2].

Neutralizing anti‐Spike protein monoclonal antibodies (mAbs) have demonstrated to be effective for treatment of mild or moderate COVID‐19 in patients with high risk of developing severe disease [5]. For this reason, in November 2020, emergency use of bamlanivimab (LY‐CoV555, Eli Lilly and Company), bamlanivimab in association with etesevimab (LY‐CoV016, Eli Lilly and Company), and casirivimab/imdevimab (REGNCOV2, Regeneron Pharmaceuticals) was approved by the US Food and Drug Administration [6, 7]. Similarly, Italian medicine agency (Agenzia Italiana del Farmaco, AIFA) approved the use of these mAbs for outpatient treatments or treatment of patients hospitalized for other reasons than COVID‐19. To date, little is known on the efficacy of mAbs in patients with HM especially among those with healthcare‐associated COVID‐19 occurring while hospitalized and undergoing chemotherapy.

The aim of our study is to investigate outcome of hospital‐acquired SARS‐CoV‐2 infection and impact of an early treatment with mAbs on the course of COVID‐19 in patients hospitalized for HM.

## 2 METHODS

### 2.1 Study design

We conducted a retrospective multicenter cohort study including all adult patients with HM who acquired SARS‐CoV‐2 infection during hospitalization in the period from November 2020 to December 2021.

Patients were enrolled in four Italian teaching hospitals: Istituto di Ricovero e Cura a Carattere Scientifico (IRCCS) Sant'Orsola Hospital, Bologna; IRCCS San Raffaele Hospital, Milan; IRCCS Ospedale Policlinico San Martino, Genoa; Policlinico Federico II, Naples. The study was approved by Ethics Committee of Area Vasta Emilia Centrale (n. 283/2020/Oss/AOUBo).

### 2.2 Criteria for SARS‐CoV‐2 screening and definitions

In the participating hospitals routinary screening for SARS‐CoV2 was performed in all admitted patients at admission, usually once weekly in asymptomatic patients, in case of other nosocomial cases and in case of symptoms suspicious for COVID‐19 using nasal swab for real‐time polymerase chain reaction (RT‐PCR) assay.

SARS‐CoV‐2 infection was defined on a new laboratory‐positive infection detected by RT‐PCR assay in respiratory specimen. Infection onset was set on the day of first SARS‐CoV‐2 RT‐PCR detection.

Hospital‐acquired SARS‐CoV‐2 infection was defined by a new onset of COVID‐19 symptoms, and positive result of SARS‐CoV‐2 molecular test occurred at least 14 days after hospital admission [9] or 7 days in case of documented in‐hospital exposure to another diagnosed case of COVID‐19, in patients with one or more negative RT‐PCR test performed at hospital admission or afterwards. We considered the time‐lapse of 14 days as the maximum estimated period of viral incubation as reported in previous studies [8] and according with last ECDC surveillance definitions [9].

We chose the study population of hematologic cancer patients who acquired SARS‐CoV‐2 infection during hospitalization because they represent the population most vulnerable to have severe COVID‐19. Moreover, in this way we were able to provide a homogeneous control group of nontreated patients followed from infection onset and excluding outpatients who have generally been tested at different time‐points of the course of infection.

### 2.3 Endpoint and exposures

The main exposure variable was administration of mAbs. Choice of mAbs as compound of COVID‐19 treatment was basically driven by the availability of the drug at the moment of the infection. As use of mAbs has been authorized in Italy from March 2021, the group of patients who did not receive mAbs consisted with who acquired infection before that period. Indeed, as nontreated patients acquired infection during the second COVID‐19 wave (from November 2020), analysis of their outcome should not be biased by the rapid advances in care that had characterized the first months of epidemics. The primary endpoint was a clinical failure defined as composite outcome of mortality and/or invasive and noninvasive ventilation (NIV) within 90 days from infection onset. We also assessed 30‐day mortality.

Additionally, we collected data such as age, gender, main comorbidities using Charlson comorbidity index [10], underlying hematological disease and disease status, concomitant treatments; COVID‐19 severity defined by WHO criteria [11], other COVID‐19 treatments.

### 2.4 Statistical analysis

In order to test our hypothesis that an early treatment with mAbs could be beneficial in terms of survival, we compared outcomes in patients with hospital‐acquired SARS‐CoV‐2 infection considering the administration of bamlanivimab, bamlanivimab/etesevimab or casirivimab/imdevimab as the main exposure variable.

Patient characteristics and clinical features were analyzed as categorical variables, presented as absolute numbers and their relative frequencies, and continuous variables, presented as the mean and standard deviation if normally distributed or as the median and interquartile range (IQR) if non‐normally distributed. The normal distribution of data was assessed with the Shapiro–Wilk and Kolmogorov–Smirnov tests. Clinical features and variables were compared between groups using the Mann–Whitney *U*‐test and the chi‐squared test (or Fisher's exact test when appropriate) for continuous and categorical variables, respectively. A *p*‐value ≤ 0.05 was considered statistically significant. The primary endpoint was evaluated as follows. First Kaplan–Meier's curves were used to assess the cumulative percentage of composite outcome among participants receiving or mAbs or other treatments. Second, variables associated with composite outcome at univariate analysis (*p* < 0.1) were introduced into Cox regression multivariable model. Patients with incomplete follow‐up were censored using the maximal follow‐up time. The time to composite outcome was calculated from the day of infection to mortality, NIV or invasive ventilation, whichever came first. Other exploratory analyses were performed with similar approach to assessing 30‐day mortality. All the analyses were performed using the Statistical Package for Social Sciences (SPSS for Windows version 21.0, SPSS Inc., Chicago, IL, USA).

## 3 RESULTS

During the study period, 53 patients with HM and hospital‐acquired SARS‐CoV2 infection were included. All patients were admitted for chemotherapy; the median time from admission to first positive swab was 14 (IQR 9–23) days. In all cases patients had one or more negative molecular test performed between admission and the index positive test. One patient died within 48 h after the diagnosis of infection and was excluded. Thus, 52 patients were analyzed. Of these, 29 (60%) were male, and the median (IQR) age was 62 (48–70). The most common underlying HMs were non‐Hodgkin lymphoma (18, 37%), acute myeloid leukemia (14, 29%), multiple myeloma (7, 15%). Forty‐five (86%) patients were on treatment with chemotherapy or received chemotherapy in the last 30 days, that was started with a median (IQR) of 13 (7–20) days before SARS‐CoV2 infection.

Twelve (23%) received at least one dose of anti‐SARS‐CoV2 vaccine at least 14 days before SARS‐CoV‐2 infection or earlier, five (10%) received two doses, and none received three doses. In all cases mRNA vaccine was administered.

Overall, 19 patients received treatment with mAbs after a median (IQR) of 1 (1–1) days from positive RT‐PCR assay reporting SARS‐CoV2 infection. Casirivimab/imdevimab was administered in 10 (53%) patients, five received 1200 mg/1200 mg dosage and five 600 mg/600 mg dosage. Bamlanivimab/etesevimab 700 mg/1400 mg was administered in eight and Bamlanivimab monotherapy 700 mg in one patient. Characteristics of patients who received mAbs and not are shown in Table 1.

**TABLE 1:** Characteristics, management, and outcomes of patients treated with or without mAbs

	**Total *N* = 52**	**Patients treated with mAbs *N* = 19**	**Patients treated without mAbs *N* = 33**	***p‐Value***
*Characteristics at SARS‐CoV‐2 diagnosis*				
**Age, years, median (IQR)**	62 (48–709	60 (47.75–66)	63 (51–71)	0.69
**Male sex *n* (%)**	32 (61.5)	14 (73.3%)	18 (54.5)	0.24
**Charlson comorbidity index, median (IQR)**	4 (2–5)	4 (2–5.75)	4 (2–5)	0.87
**Anti‐SARS‐CoV‐2 vaccination, *n* (%)**	12 (25)	12 (63)	0 (0)	<0.001
**I dose**	7 (13)	7 (36)	0	
**II doses**	5 (9)	5 (26)	0 (0)	0.02
**III doses**	0 (0)	0 (0)	0 (0)	
**Time from last vaccine dose to infection**				
**0–3 months**	2 (4)	2 (10)	0 (0)	0.19
**3–6 months**	2 (4)	2 (10)	0 (0)	0.19
**>6 months**	8 (42)	8 (42)	0 (0)	<0.001
**Anti‐SARS‐CoV2 serology tested, *n* (%)**	23 (44.2)	11 (57.9)	12 (36.4)	
• **Seropositive anti‐S, *n* (%)**	9 (39.1)	9 (81.8)	0 (0)	<0.001
**WBC count, median (IQR)**	3420 (470–7590)	2485 (200)	3700 (585)	0.52
**ANC count, median (IQR)**	1550 (135–4976)	1105 (15–4605)	1600 (185–5600)	0.84
*Hematologic malignancy*				0.95
**Non‐Hodgkin lymphoma, *n* (%)**	20 (38)	7 (37)	13 (39)	
**Hodgkin lymphoma, *n* (%)**	3 (6)	1 (5)	2 (6)	
**Acute myeloid leukemia, *n* (%)**	15 (29)	9 (27)	6 (32)	
**Acute lymphoblastic leukemia, *n* (%)**	4 (8)	2 (10)	2 (6)	
**Multiple myeloma, *n* (%)**	8 (15)	3 (16)	5 (15)	
**Chronic lymphocytic leukemia**	1 (2)	0 (0)	1 (3)	
**Previous HSCT, *n* (%)**	8 (15.4)	1 (5.3)	7 (21.2)	0.23
**GVHD, *n* (%)**	2 (4)	1 (5.6)	1 (3.1)	1
*Hematologic disease status*				0.8
**Progressive disease, *n* (%)**	15 (28.8)	4 (21.1)	11 (33.3)	
**Disease onset, *n* (%)**	26 (50)	10 (52.6)	15 (45.5)	
**Partial response, *n* (%)**	2 (3.8)	1 (5.3)	1 (3)	
**Complete response, *n* (%)**	6 (11.5)	2 (10.5)	4 (12.1)	
**Stable disease, *n* (%)**	3 (5.8)	1 (5.3)	2 (6.1)	
*Reason for hospital admission*				0.46
**Induction CHT, *n* (%)**	14	5 (26.3)	9 (27.3)	
**Consolidation CHT, *n* (%)**	9	4 (21.1)	5 (15.2)	
**Salvage CHT, *n* (%)**	5	2 (10.5)	3 (9.1)	
**HSCT, *n* (%)**	8	1 (0)	7 (12.1)	0.35
• **Allogeneic, n (%)**	3/8 (37))	1/1(100)	2/7 (29)	
• **Autologous, *n* (%)**	5/8 (62)	0 (0)	5/7 (71)	
**CAR‐T therapy, *n* (%)**	1 (1.9)	1 (5.3)	0 (0)	
**Other reason, *n* (%)**	18	6 (31.6)	12 (36.4)	
*Treatment received*				
**Dexamethasone, *n* (%)**	31 (59.6)	9 (47.4)	22 (66.7)	0.24
**Remdesivir, *n* (%)**	20 (38.5)	3 (15.8)	17 (51.5)	0.017
**Convalescent plasma, *n* (%)**	10 (19.2)	0 (0)	10 (30.3)	0.009
**Tocilizumab, *n* (%)**	3 (6.4)	1 (7.1)	2 (6.1)	1
**Ruxolitinib, *n* (%)**	2 (3.9)	0 (0)2	2 (6.2)	0.52
*Clinical evolution (90 days)*				
**Oxygen therapy, *n* (%)**	31 (62)	10 (52.6)	21 (67.7)	0.37
**NIV, *n* (%)**	15 (28.8)	1 (5.3)	14 (42.4)	0.004
**HFNC, *n* (%)**	7 (16.3)	2 (13.3)	5 (17.9)	1
**IOT, *n* (%)**	8 (15.7)	1 (5.3)	7 (21.9)	0.23
**ECMO, *n* (%)**	1 (2)	0 (0)	1 (3.1)	1
**CVVH, *n* (%)**	1 (2)	0 (0)	1 (3.1)	1
**30‐day mortality, *n* (%)**	14 (26.9)	3 (15.8)	11 (33.3)	0.2
**COVID‐19‐related 30‐day mortality, *n* (%)**	11 (21)	1 (5)	10 (30)	0.04
**90‐day mortality *n* (%)**	19 (36.5)	4 (21.1)	15 (45.5)	0.13

Abbreviations: ANC, absolute neutrophil count; CAR‐T, chimeric antigen receptor T cell therapies; CHT, chemotherapy; CVVH, continuous veno‐venous hemofiltration; ECMO, extracorporeal membrane oxygenation; HFNC, high‐flow nasal canula; HSCT, hematopoietic stem cell transplant; IQR interquartile range; mAb, monoclonal antibodies; NIV, noninvasive ventilation; OTI, orotracheal intubation; WBC, white blood cell.

At the diagnosis of SARS‐CoV‐2 infection patients were asymptomatic in 40% of cases (42% in patients receiving mAbs vs. 39% in the non‐mAbs group, *p* = 0.84) and had mild symptoms in 60% of cases.

### 3.1 Primary outcome

**FIGURE 1:**
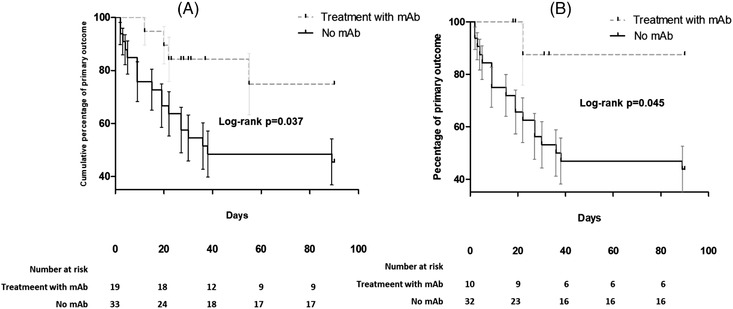
Kaplan–Meier curves showing differences in primary outcome (composite of mortality or need for ventilation within 90 days from infection onset) between hematologic patients treated or not with antispike monoclonal antibodies in the whole cohort (panel A) or after excluding patients who were antispike Ab seropositive before administration of monoclonal antibodies

During the follow‐up, the primary outcome occurred in 22 (42%) of patients. More specifically, 15 (29%) patients needed NIV, 8 (15%) mechanical ventilation (MV), and 19 (36%) died within 90 days. The rate of patients with primary outcome was 21% (4/19) in patients receiving mAbs versus 54% (18/33) in the non‐mAbs group (log‐rank *p* = 0.03. Figure 1A). Results were consistent after excluding patients with baseline positive serum antispike antibodies collected before mAbs administration (log‐rank *p* = 0.06, Figure 1B).

**FIGURE 2:**
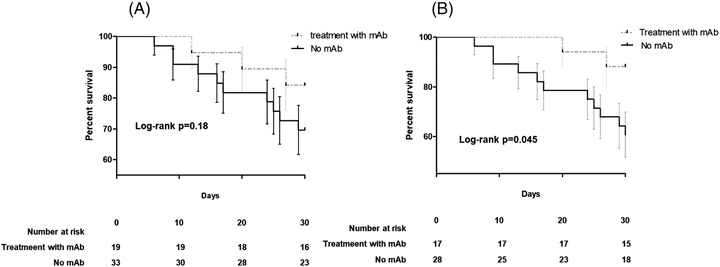
Kaplan–Meier curves showing difference in 30‐mortality rate among hematologic patients receiving monoclonal antispike antibodies for the treatment of SARS‐CoV‐2 infection in the whole cohort (Panel A, 52 patients) or in patients undergoing chemotherapy (Panel B, 45 patients)

After comparison between patients with and without clinical failure (Table 2), the former group were more likely to be older (median [IQR] 69 [61–72] vs. 58 [46–66], *p* = 0.001), had higher age‐adjusted Charlson comorbidity index (median [IQR], 5 [3.25–5] vs. 3 [2–5], *p* = 0.002) than the latter group. At multivariable analysis using Cox regression model (Table 3) administration of mAbs was independently associated with a lower rate of clinical failure (HR 0.11 [95% CI 0.01–0.85, *p* = 0.01]) after adjusting for disease status, age‐adjusted Charlson comorbidity index, and complete anti‐SARS‐CoV‐2 vaccination (at least two doses) before the disease onset.

**TABLE 2:** Characteristics and management of patients who met and did not meet the composite outcome of clinical failure (90‐day death or NIV or MV)

	**Patients with clinical failure, n = 22**	**Patients without clinical failure, n = 30**	***p‐*Value**
*Characteristics at COVID‐19 diagnosis*			
**Age (year), median (IQR)**	69 (61.25–72)	58 (46–66)	0.001
**Male sex *n* (%)**	13 (59.1)	19 (63.3)	0.78
**Charlson comorbidity index, median (IQR)**	5 (3.25–5)	3 (2–5)	0.002
**HSCT, *n* (%)**	3 (13.6)	5 (16.7)	1
**GVHD, *n* (%)**	1 (4.8)	1 (3.4)	1
**Complete anti‐SARS‐CoV‐2 vaccination, *n* (%)**	3 (13.6)	10 (33.3)	0.19
**Anti‐SARS‐CoV2 serology tested, *n* (%)**	11 (50)	12 (40)	
**Seropositive anti‐S, *n* (%)**	3 (27.3)	6 (50)	0.4
**WBC count, median (IQR)**	4070 (442.5–8540)	2890 (535–7020)	0.46
**ANC count, median (IQR)**	1270 (195–3537.5)	1600 (55–5305)	0.65
*Hematologic malignancy*			0.68
**Lymphoma**	8 (36)	14 (47)	
**Acute Leukemia**	10 (45)	9 (30)	
**Multiple myeloma, *n* (%)**	3 (14)	6 (20)	
**Other**	1 (4)	1 (3)	
*Hematologic disease status*			0.93
**Progressive disease, *n* (%)**	7 (31.8)	8 (26.5)	
**Disease onset, *n* (%)**	11 (50)	15 (50)	
**Partial response, *n* (%)**	1 (4.5)	1 (3.3)	
**Complete response, *n* (%)**	2 (9.1)	4 (13.3)	
**Stable disease*, n* (%)**	1 (4.5)	2 (6.7)	
*Reason for hospital admission*			0.46
**Consolidation CHT, *n* (%)**	3 (13.6)	6 (20)	
**Salvage CHT, *n* (%)**	4 (18.2)	1 (3.3)	
**Induction CHT, *n* (%)**	5 (22.7)	9 (30)	
**HSCT, *n* (%)**	5 (16)	3 (14)	
**CAR‐T therapy, *n* (%)**	0 (0)	1 (3.3)	
**Other reason, *n* (%)**	8 (36.4)	10 (33.3)	
*COVID‐19 management*			
**Corticosteroids, n (%)**	19 (86.4)	12 (40)	0.001
**Indication: underlying disease, n (%)**	7 (31)	7 (23)	0.49
**Indication: severe COVID‐19, n (%)**	11 (50)	4 (13)	0.004
**Remdesivir, *n* (%)**	11 (50)	9 (30)	0.16
**Convalescent plasma, *n* (%)**	5 (22.7)	5 (16.7)	0.73
Early plasma treatment*, *n* (%)	3 (13.6)	4 (13.3)	0.97
**Ruxolitinib, *n* (%)**	2 (9.5)	0 (0)	0.16
**Tocilizumab, *n* (%)**	2 (9.1)	1 (4)	0.59
**Early mAb, *n* (%)**	4 (18.2)	15 (50)	0.023
**Oxygen therapy, *n* (%)**	20 (95.2)	11 (37.9)	<0.001
**HFNC, *n* (%)**	6 (33.3)	1 (4)	0.015
**NIV, *n* (%)**	15 (68.2)	0 (0)	<0.001
**MV *n* (%)**	8 (36.4)	0 (0)	0.001
**ECMO, n (%)**	1 (4.5)	0 (0)	0.44
**CVVH, *n* (%)**	1 (4.8)	0 (0)	0.42

Abbreviations: ANC, absolute neutrophil count; CAR‐T, Chimeric Antigen Receptor T cell therapies; CHT, chemotherapy; CVVH, continuous veno‐venous hemofiltration; ECMO, extracorporeal membrane oxygenation; HFNC, high‐flow nasal canula; HSCT, hematopoietic stem cell transplant; IQR, interquartile range; mAb, monoclonal antibodies; MV, mechanical ventilation; NIV, noninvasive ventilation; WBC, white blood cell.

*Administered within 7 days from first positive RT‐PCR

**TABLE 3:** Multivariable Cox regression model for primary outcome (composite of mortality or need for ventilation within 90 days from infection onset) in patients with hematologic malignancies and hospital‐acquired SARS‐CoV‐2

**Model covariate**	**Hazard ratio**	**95% CI**	***p‐Value***
Age‐adjusted Charlson comorbidity index	1.18	0.98–1.17	0.07
Hospital admission for salvage chemotherapy	3.98	1.16–13.36	0.028
Treatment with monoclonal antispike protein antibodies	0.11	0.01–0.85	0.01
Previous anti‐SARS‐CoV‐2 vaccination (two doses)	5.13	0.44–59.3	0.19

### 3.2 Impact of mAbs on 30‐day mortality

Overall, 14 (27%) died within 30 days from COVID‐19 onset of which three (15%) in the mAbs group and 11 (33%) in the control group, with no statistical differences (log‐rank *p* = 0.18) Figure 2. When the analysis was restricted to 45 patients who received chemotherapy in the previous 30 days, there was lower mortality in patients treated with mAbs versus non‐mAbs group (2/17 [12%] vs. 11/29 [39%], *p* = 0.05). Similarly, in this group, mAbs was independently associated with survival at Cox regression model for 30‐day mortality (HR 0.11 [95% CI 0.01–0.89] *p* = 0.034) after adjustment for underlying disease and status. In 11 cases of death occurring within 30 days from SARS‐CoV‐2 diagnosis among patients not receiving mAbs, 10 were judged as consequences of COVID‐19. On the other hand, among three deaths occurring within 30 days in patients treated with mAbs, none was attributed to COVID‐19 but all to the underlying HM. In fact, none of these patients was receiving oxygen or had symptoms of COVID‐19 at the time of death.

### 3.3 Viral Shedding

Time to negative nasal swab was calculated from the first positive sample to the first negative sample of two consecutive negative nasal swabs. This information was available only in 22 patients, of which seven treated with mAbs, Overall, the viral shedding was 48 (17–102) days in this population. Patients treated with mAbs had significantly shorter viral shedding (median [IQR] 12 days [4–43]) when compared to patients from non‐mAbs group (median [IQR] 77 days [43–156], *p* = 0.003).

## 4 DISCUSSION

In this study, we report the clinical features and outcome of 52 hematologic cancer patients with hospital‐acquired SARS‐CoV‐2 infection. Primary outcome of mortality or need for invasive or noninvasive ventilation within 90 days from infection onset occurred in 42% of patients; particularly 37% died, and 42% underwent NIV or MV.

Our results evidenced an increased risk of poor outcome of hospital‐acquired SARS‐CoV‐2 infection in patients with HM undergoing chemotherapy, which is even worse than current epidemiologic data on hematologic cancer patients published by Pagano et al. reporting 25% overall mortality rate (in particular, about half of the study population had received chemotherapy in the previous month) [10]. It should be noted that most of reports on hematologic patients with COVID‐19 gathered mainly community patients with stable disease or in remission, who are less immunocompromised than those undergoing active chemotherapy. On the contrary, our study focused on patients hospitalized for active malignancy of whom 86% with ongoing or very recent chemotherapy, and 53% had severe neutropenia at infection onset. Therefore, this is a subpopulation of HM patients with potentially the most catastrophic consequences of nosocomial SARS‐CoV‐2 infection. Indeed, a multicenter study, which focused on outcome of hospital‐acquired compared to community‐acquired SARS‐CoV‐2 infection in cancer patients, showed an important difference in 14‐day survival (78% versus 53%, respectively). Moreover, mortality rate in the subgroup of patients with HM and hospital‐acquired infection was 75% [8]. These data raise awareness on very high probability of unfavorable course of nosocomial SARS‐CoV‐2 infection in this subgroup of severely immunocompromised patients [10]. Additionally, the impact might be worsened if prolonged SARS‐CoV‐2 infection delays chemotherapy cycles further worsening patient's chances of survival.

Another important finding of our study was that an early use of mAbs was associated with lower rate of critical COVID‐19^11^ (21% versus 54% in patients treated and not with mAbs, respectively) even after adjustment for potential confounders such as age or comorbidities. Although clinical trials with mAbs have demonstrated their efficacy in the population with high risk to develop severe disease, including immunocompromised patients, studies focusing on outcome of hematologic cancer patients are currently limited and mainly consist of case reports [12, 13, 14]. In a single‐center retrospective study, 38 patients with HM received bamlanivimab or casirivimab/imdevimab, and four of them (11%, all who received bamlanivimab monotherapy) needed hospitalization, two of them (5%) developed severe COVID‐19, and one died [15]. Another study assessed outcomes of 38 patients with active cancer (18/38 with HM) treated with mAbs (bamlanivimab or casirivimab/imdevimab). Among them, nearly 8% required hospitalization within a median of 25 days (IQR 5–29) after mAb administration whereas 5% died from complications related to COVID‐19 [16]. Finally, a Czech multicenter study assessed efficacy of early mAbs in 88 hematologic patients with COVID‐19. Progression to severe/critical disease and COVID‐19‐related deaths occurred in 17% and 8% of cases, respectively; compared to a control cohort of 575 hematologic patients with COVID‐19 who did not receive any specific anti‐SARS‐CoV‐2 therapy, mortality was significantly lower in a subgroup of 69 mAb‐treated and remdesivir/convalescent plasma‐untreated patients (6% vs. 16%, *p* = 0.002) [17]. However, none of these cohorts specifically reported outcomes on the use of mAbs targeting patients actively undergoing chemotherapy.

In our study, interestingly, a significantly lower mortality associated with the use of mAbs was noticed in the subgroup of patients undergoing chemotherapy during the previous 30 days. In addition, use of mAbs in this group was an independent protective factor against 30‐day mortality. As mentioned before, concomitant chemotherapy for HM is supposed to worsen outcome in patients with COVID‐19; therefore prompt intervention with mAbs at infection onset may be crucial to arrest progression to severe COVID‐19.

We observed that therapy with mAbs was mostly administered within 1 day after diagnosis. Such a prompt diagnosis and treatment of SARS‐CoV‐2 infection may have an impact on clinical outcome as early administration of therapies targeting viral phase is considered a cornerstone of COVID‐19 treatment. Indeed, mAbs have shown efficacy in outpatients with mild/moderate COVID‐19 within 5 days from symptoms onset, although benefits appear limited in hospitalized patients with more advanced disease [18]. In our study most patients had no or only mild symptoms (fever, asthenia, etc.), which in this population could be confused with symptoms due to underlying disease, treatment, or other infection. Therefore, implementation of a hospital‐wide surveillance system with molecular swabs performed at admission and periodically during hospitalization has provided the possibility to early diagnose SARS‐CoV‐2 infection enabling immediate administration of COVID‐19 therapies.

Finally, our results showed that patients receiving mAbs had a significant shorter viral shedding compared to non‐mAbs treated patients. Other studies showed that viral shedding in immunocompromised host could be much longer that immunocompetent patients [3, 19]. As hematologic treatments are generally interrupted during active infections, shortening of viral shedding with mAbs may allow an early restarting of chemotherapy, which is a determinant factor for survival in patients with HM.

This study has some limitations. First, the small sample size may limit the generalizability of results, but luckily nosocomial cases of SARS‐CoV2‐ infection remain infrequent, and currently most data come from case reports. In addition, typically for SARS‐CoV‐2 pandemics, patients’ outcomes have progressively improved over time due to earlier testing and progressively introduced treatment options and vaccines. However, in hospitalized HM patients, SARS‐CoV‐2 was diagnosed early from the beginning of pandemics due to weekly testing introduced at the beginning of the pandemic, and the use of antiviral treatment was quite frequent with 50% of patients treated in the non‐mAbs group. Moreover, previous vaccination in mAbs group may have had an additional positive impact on outcome, and even though only 9% of patients had attained complete vaccination with two doses before infection, all of them were in mAbs group. Nevertheless, significant impact of mAbs on survival was present even after excluding patients with positive anti‐SARS‐CoV‐2 serology at baseline. Finally, efficacy of different mAbs is strictly dependent on the prevalence of circulating SARS‐CoV‐2 viral variants neutralized by given mAbs. Subsequently, the circulation of the Omicron variant has compromised the efficacy of bamlanivimab/etesevimab and casirivimab/imdevimab leaving sotrovimab as the only available option. However, the Omicron variant has also other characteristic of apparently lower virulence. Therefore, our study in the pre‐Omicron era provided us with a homogenous population, in which the assessment of the severity of disease is probably not biased by viral variants.

In conclusion, burden of hospital‐acquired SARS‐CoV‐2 infection in patients with HM is currently relevant and may significantly affect prognosis in this group. Early treatment with mAbs appears to be effective in preventing COVID‐19 progression even in the most vulnerable categories.

## AUTHOR CONTRIBUTIONS

LB and MB performed research and wrote the manuscript. DT, BT, CO, CS, SM, CC, FT, ZP, and EZ performed data collection. MB performed data analysis. MM, MB, PV, and MB supervised the study. All Authors reviewed and approved the manuscript.

## CONFLICT OF INTEREST

The authors declare that there is no conflict of interest that could be perceived as prejudicing the impartiality of the research reported.

## FUNDING INFORMATION

Open Access funding provided by IRCCS Ospedale Policlinico San Martino.

[Correction added on 28 November 2022, after first online publication: BIBLIOSAN funding statement has been added.]

## ETHIC STATEMENT

The study was conducted in accordance with the current version of the Declaration of Helsinki, the International Conference on Harmonization Good Clinical Practice (ICH‐GCP), and national legislation for data protection.

## Data Availability

The data that support the findings of this study are available upon request from the corresponding author. The data are not publicly available due to privacy or ethical restrictions.
